# Population dynamics of *Phaius flavus* in southeast China: Reproductive strategies and plants conservation

**DOI:** 10.1371/journal.pone.0272929

**Published:** 2022-08-15

**Authors:** Jun Li, Ya-ting Zhu, Lun-yan Chen, Ai-xian Lu, Hong-yu Ji, Hai-ping Liu, Ze-xin Li, Zuo-dong Lin, Sha-sha Wu, Jun-wen Zhai

**Affiliations:** 1 College of Landscape Architecture, Fujian Agriculture and Forestry University, Fuzhou, Fujian Province, China; 2 National Forestry and Grassland Administration for Orchid Conservation and Utilization, Fujian Agriculture and Forestry University, Fuzhou, Fujian Province, China; Shandong University, CHINA

## Abstract

Because of species diversity and troubling conservation status in the wild, Orchidaceae has been one of the taxa with most concern in population ecological research for a long time. Although Orchidaceae is a group with high adaptability, they have become endangered for complex and various reasons such as the germination? difficulty and habitat loss, which makes it difficult to develop an accurate protection strategy. *Phaius flavus* is a terrestrial orchid which used to be widely distributed in central and southern Asia; however, large populations are difficult to find in the wild. Thus, the aim of this study was to provide a new perspective for conserving endangered *P*. *flavus* by investigating the mechanisms of its population decline; we established time-specific life and fertility tables, age pyramids, survival curves, and mortality curves for this plant and then conducted Leslie matrix model. We found that both of the populations from Wuhu Mount (WM) and Luohan Mount (LM) showed declining trends and exhibited pot-shaped age pyramids, low net reproductive rates, and negative intrinsic growth rates. The population from the Beikengding Mount (BM) showed a stable status with a bell-shaped age pyramid. However, it has a significant risk of decline because of the low net reproductive rate and intrinsic growth rate. This study use time-specific life and fertility tables, age pyramids, survival curves, and mortality curves, showed that the population decline of *P*. *flavus* could be attributed to 1) the shortage of seedlings caused by the low germination rate in the wild and 2) the loss of adult individuals caused by anthropogenic disturbances. To protect this species from extinction in these areas, we suggest that human activities in these habitats should be strictly forbidden and *ex situ* conservation of this plant in botanical gardens is also necessary.

## 1. Introduction

Organisms adjust their life history traits and reproductive strategies to adapt to the environmental conditions of their locations [1]. Plants face a variety of risks during their lifetimes, such as competition, predation, diseases and pests. To ensure their reproduction, species in different habitats have evolved a variety of population adaptation strategies [2]. The study of population dynamics can reflect the characteristics of quantitative variation, living status, and habitat fitness [3]. Gui *et al*. studied a population of *Phoebe bournei* in central Jiangxi Province and, based on survival curves and quantitative analysis, found its population was capable of self-development but was sensitive to external disturbances [4]. Zhu et al. analyzed the quantity characteristics of *Ginkgo biloba* by investigating population inventories and distributions, and argued that this plant had a long period what? under severe fragmentation of its distribution, and suggested improving its habitat quality and strengthening protections [5]. Xiao et al. studied a population of *Michelia wilsonii*, which is a kind of PSESP (Plants Species with Extremely Small Populations), in Ya-an and found a sharp decrease of the population in the early age period, stability in the middle age period, and a decline in the late age period [6]. The above studies show that quantitative analysis is a reliable reference to understand the living status of plant populations, and the findings would be helpful for future preservation strategy development.

Orchidaceae is one of the largest and most diverse flowering plant families and consists of approximately 800 genera and 27,800 species [7]. Orchids live in nearly every terrestrial ecosystem in the world except for polar regions and arid regions such as deserts, thus they are usually considered to be highly adaptive to various environments and particularly diversified in tropical areas [8]. However, the survival of wild orchids faces challenges due to various factors [9]. Thus, as a flagship group in plant conservation, orchids have been a hot topic in endangered plant conservation research for a long time. First, habitat loss is the most lethal variable for explaining extinction probabilities [10]. Human activities (including forest clear-cutting and the expansion of agricultural landscapes) have exacerbated the natural fragmentation of landscapes [11]. Habitat fragmentation decreases plant population sizes and increases spatial isolation, which hampers the exchange of seeds and pollen between fragmented populations [12]. Second, the physiological adaption of orchids makes them sensitive to changes in temperature and rainfall. Especially for perennial orchids, climate change may affect different parts of the life cycle, including germination [13], growth [14], flowering [15, 16], and survival [17]. Third, orchid survival has close associations with the biotic environment. Orchids have complicated pollination systems with specialized pollinators, and their survival strongly depends on pollination success [16, 18]. At the same time, the entire Orchidaceae family appears to consist of mycorrhizal “cheaters,” as orchids have short-circuited the reciprocal plant-fungal exchange [19]. The distribution and availability of suitable mycorrhiza usually determine the seeding recruitment of orchids, and successful seeding establishment is a critical life story stage [20]. However, we lack information necessary for the conservation of Orchidaceae till now, especially for species that are known to be endangered [16].

*Phaius flavus* (Bl.) Lindl. is a tall, tuberous, perennial orchid that is mainly distributed in Asia, including China, Japan, India and most Southeast Asian countries [21]. It grows on damp slopes under trees at altitudes from 300 to 2,500 m and has a long history of cultivation as an ornamental and medicinal plant in China. As scaled artificial cultivation systems have not been established and extensively applied, wild populations of *P*. *flavus* have been excessively harvested over a long period as commercial demand has increased. Meanwhile, habitat fragmentation caused by human activities has made it increasingly difficult for *P*. *flavus* populations to survive in the wild. As a result, it is now difficult to find large populations in the field. Wang et al. studied the tissue culture and aseptic germination of *P*. *flavus* [22]. They provided a technology to mass produce *P*. *flavus* seedlings, which has potential application prospects for the protection and development of *P*. *flavus*. Kawakami et al. isolated a flexuous virus from cultivated *P*. *flavus* in Japan and analyzed its complete nucleotide sequence, and the results showed that its open reading frames differed from those of previously reported potex viruses [23]. The flora of Chinese orchids are distinguished by rich diversity in geographical types, especially in the broad subtropical area between the Qinling Mountains and the Tropic of Cancer [24]. In China, the main distribution areas of *P*. *flavus* are in the subtropical monsoon climate zone, which is characterized by high temperatures and abundant rainfall in summer with warm and dry winters. It is a representative of wild *P*. *flavus* populations in Fujian because it is located in the southeast part of China and is in a typical zone of the subtropical monsoon climate.

Here, population dynamics and individual number predictions were studied in three populations of *P*. *flavus* in Fujian Province based on an investigation of its biological characteristics. The following questions were addressed: 1) What is the survival status for wild *P*. *flavus*? If the answer is not optimistic, what is the possible reason for this situation? 2) Is there a recruitment situation and development tendency of the three populations studied here? Additionally, if there is a gap of data between three populations, how did the gap come about? By solving these questions, we discuss the growth and reproduction of *P*. *flavus* and provide a theoretical basis for the conservation of orchid germplasm resources.

## 2 Materials and methods

### 2.1 Study sites

This study was conducted on three natural populations of *P*. *flavus* located in Fujian, China, namely, the Wuhu Mount population (WM population), the Luohan Mount population (LM population) and the Beikengding Mount population (BM population). The WM population is present in the Sandiejing National Forest Park in the western part of Fuzhou, southern subtropical region, while the LM and BM populations are present in Sanming City, central subtropical region [25]. Other information for these sites is shown in Table 1. The dominant species in these study areas are *Sloanea sinensis*, *Castanopsis sclerophylla*, *Schima superba*, *Ficus erecta*, *Aspidistra elatior*, *Oplismenus undulatifolius*, *Colysis elliptica* and *Gardneria multiflora*.

**Table 1:** Survey of study site.

Name of study site	Wuhu Mount	Luohan Mount	Beikengding Mount
Climate zone	Southern subtropical monsoon	Central subtropical monsoon	Central subtropical monsoon
Soil type	Lateritic red soil, red soil	Red soil, yellow red soil	Red soil, yellow red soil
Longitude (E)	118°52′10″~119°25′31″	118°9′58~118°11′28″	118°6′14~118°25′22″
Latitude (N)	25°47′35″~26°36′28″	25°59′35″~25°58′44″	26°21′49″~26°20′21″
Altitude (m)	18~611	170~1287.5	198~1220
Average temperature (°C)	19.5	19.8	19.6
Average annual precipitation (mm)	1759	1445	1700
Annual sunshine duration (h)	2028	1762.8	1864.8
Frostless season (d)	330	298	288

### 2.2 Investigation of biological characteristics

Three transects were established along three valleys with the distribution of *P*. *flavus*. Based on a complete survey along these transects, we defined one site each in Wuhu Mount and Luohan Mount and two in Beikengding Mount. Because the distance between the two study sites in Beikengding Mount was less than 1 km and there were no habitat breaks or barriers between individuals, all *P*. *flavus plants* at that location were collectively placed into the BM population. Each transect consisted of adjacent 50 cm × 50 cm plots. A total of 146 *P*. *flavus* plants along three transects were mapped and labeled, and a grid method was used to obtain their relative coordinates. Pseudobulbs linked by flashy rhizomes were counted as a single individual plant and genotype. These pseudobulbs consisted of leafed pseudobulbs and leafless pseudobulbs, and their numbers were recorded, separately. Because every individual produces a new pseudobulb each year, we determine the age of each individual based on the number of pseudobulbs, this method is recorded in cultivation references and some previous studies. This regularity is shared by *Trias verrucose* [26], *Cymbidium sinense* [8], *Dendrobium sinense* [27], *Calanthe tsoongiana* [28], *Phaius flavus* and many other orchids. The above studies provide more ideas for wild orchid conservation, and also provide a reference for our study. To confirm this rule, we counted the number of buds germinated by every individual between Feb. 2019 and Apr. 2019 and further analyzed these results. The numbers of infructescences and persistent fruits were recorded.

### 2.3 Data analysis

#### 2.3.1 Establishment of time-specific life tables

Pseudobulbs of *P*. *flavus* plants form a single-chain structure during the growth process. We used an approach of spatial sequences instead temporal sequences [8, 29, 30], and the specific indicators and their data processing are as follows. We defined *X* as the age of a sample in years, *a*_*x*_ as the number of plants surviving at the start of the age interval *x*, and *l*_*x*_ as the proportion of plants surviving at the start of age interval *x*.


$$l_{x}=(a_{x}÷a_{0})\times 1000$$ (1)

We then defined *d*_*x*_ as the proportion of plants that died during the age interval from *x* to *x + 1*, *q*_*x*_ as the death rate during the age interval from *x* to *x + 1*, and *K*_*x*_ as the rate of disappearance.


$$d_{x}=l_{x}-l_{x+1}$$ (2)

$$q_{x}=d_{x}÷l_{x}$$ (3)

$$K_{x}=\ln\;l_{x}-\ln\;l_{x+1}$$ (4)

*L*_*x*_ is the average number of plants that survived during the age interval *x* and *x + 1*, *T*_*x*_ is the total number of plants that survived from age interval *x*, and *e*_*x*_ is the life expectancy at the start of age interval *x*.


$$L_{x}=(l_{x}+l_{x+1})÷2$$ (5)

$$T_{x}=\sum L_{x}$$ (6)

$$e_{x}=T_{x}÷l_{x}$$ (7)

#### 2.3.2 Age pyramids, survival curves and mortality curves

Age pyramids were plotted with *l*_*x*_ as the horizontal axis and the corresponding age level (X) as the vertical axis. The survival curve was plotted with ln *l*_*x*_ as the horizontal axis and the corresponding age level (X) as the vertical axis. The mortality curve was plotted with the *q*_*x*_ as the horizontal axis and the corresponding age level (X) as the vertical axis.

#### 2.3.3 Fertility table establishment

Based on data from the time-specific life tables, specific indicators for the fertility tables and their data processing are as follows. We defined *X* as the age of a sample in years, *l*_*x*_ as the proportion of plants surviving at the start of age interval *x*, *m*_*x*_ as the average number of progeny produced by individuals in X-age, *R*_*0*_ as the net reproductive rate, *r*_*m*_ as the intrinsic rate of increase, *λ* as the increment rate per unit, and *T*_*x*_ as the average period from the birth of parents to the birth of progeny.


$$R_{0}=\sum \left(l_{x}\times m_{x}\right)$$ (8)

$$r_{m}=\ln\;R_{0}÷T$$ (9)

$$\lambda =e^{r}$$ (10)

$$T_{x}=\sum \left(X\times l_{x}\times m_{x}\right)÷\sum \left(l_{x}\times m_{x}\right)$$ (11)

#### 2.3.4 Leslie matrix construction

The Leslie matrix is one of the best-known methods for describing the growth of populations; it is used in ecology to model changes in populations of organisms over a period of time [31]. *P*_*x*_ (the sum of survival rates from age interval *x*) was calculated on the basis of survival rate (*l*_*x*_) from the specific-time life table and *f*_*x*_ (the average number of progeny that were produced by X-age adults and survived to the next age) was calculated by using data from the fertility table.


$$P_{x}=L_{x+1}÷L_{x}=(l_{x+1}+l_{x+2})÷(l_{x}+l_{x+1})$$ (12)

$$f_{x}=P_{x}\times m_{x}$$ (13)

Then, the population massive and age structure after one unit time interval were determined from the data of the previous time interval. *N*_*t*_ is the population quantity in the *t* time interval and *N*_*0*_ is the population quantity at the time of this survey.


$$N_{t+1}=M\times N_{t}=M_{t+1}\times N_{0}$$ (14)

*M* is the population projection matrix.


$$M=\begin{array}{cccccc} f_{0} & f_{1} & f_{2} & \cdots & f_{19} & f_{20}\\ P_{0} & 0 & 0 & \cdots & 0 & 0\\ 0 & P_{1} & 0 & \cdots & \vdots & \vdots \\ \vdots & \vdots & P_{2} & \cdots & \vdots & \vdots \\ \vdots & \vdots & \vdots & \cdots & \vdots & \vdots \\ 0 & 0 & 0 & \cdots & P_{19} & 0 \end{array}$$ (15)

## 3 Results and analysis

### 3.1 Biological characteristics of *P*. *flavus* plants

*P*. *flavus* plants grow in gullies and/or near creeks with deep moist humus soil, good drainage and sunlight-scattered forest slopes or stone surfaces. Their oval pseudobulbs are linked by short, fleshy stems for which pseudobulbs are 2 to 8 cm tall and 1 to 6 cm in diameter. Pseudobulbs produce 1–6 leaves at the apex, and they remain green and functional for 1–14 years; at the same time, pseudobulbs that have lost all their leaves still retain the possibility of sprouting new buds to resume their vegetative growth and reproductive process. The leaf lengths of different individuals in the population varied widely, and the adults were stable at approximately 48 cm. New pseudobulbs usually germinated yearly from the bottom of the last generation of pseudobulbs in the middle of March. The persistence of leafless pseudobulbs and the annual proliferation of new pseudobulbs worked together to form the chain-shaped *P*. *flavus* plants.

**Fig 1:**
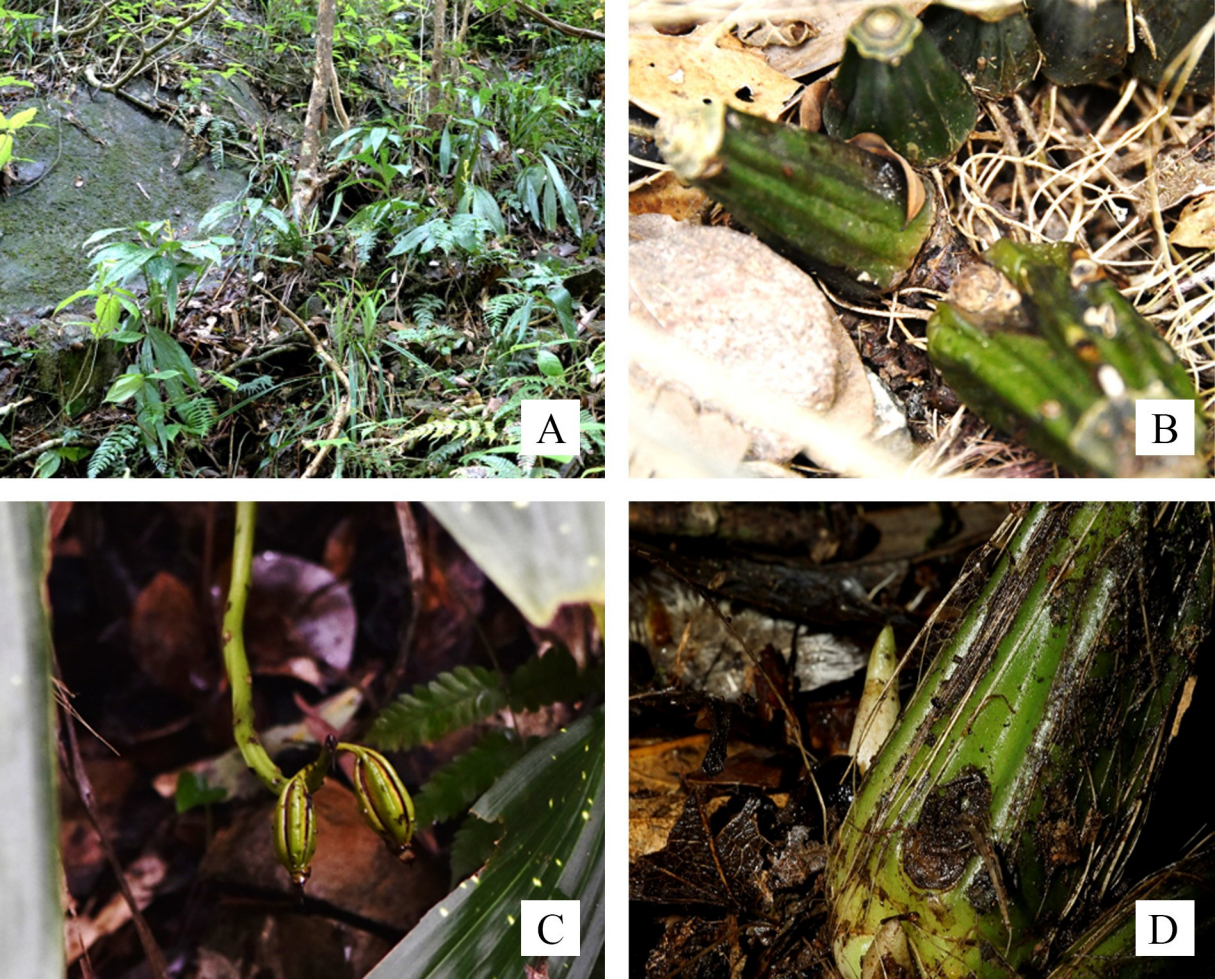
The habitat and growth characteristics of *Phaius flavus*. A. Habitat; B. pseudobulbs and fleshy stems; C. persistent dehiscent fruits; D. a budding bud.

According to our survey, the average numbers of buds that each individual generated in Feb. 2019 were 0.87 (WM population), 0.91 (LM population) and 0.83 (BM population). Based on these data, we estimate that each individual produced nearly one additional pseudobulb per year, and the number of pseudobulbs is further assumed to be this individual’s age grade. According to the statistics of persistent flower stems, *P*. *flavus* recruitments take at least 4 years from germination to flowering. Once these adult individuals enter the sexual reproduction stage, they remain fertile until their maximum lifespan has been reached. The sums of the number of persistent fruits in the 3 populations were 3 (WM population), 11 (LM population) and 50 (BM population), and the average numbers of fruits per individual in the 3 populations were 0.05 (WM population), 0.26 (LM population) and 2.63 (BM population) (Fig 1).

### 3.2 Time-specific life tables

The time-specific life tables for the 3 populations are shown in Tables 2–4. With 62 individuals and 18 age classes, the WM population is the largest but youngest population among these three, while the BM population is the smallest and oldest and has 41 individuals and 39 age classes. Abruption phenomena frequently appear for age classes over 11 and cause many formulas in the tables to have errors in the counting process. This is why there are many blanks in the tables. Especially for the BM population, there are only 9 age classes that have surviving individuals between age classes 12–39, which account for 32.1% of the total.

**Table 2:** Time-specific life table of *P*. *flavus* WM population.

*X*(year)	*a*_*x*_	*l*_*x*_	*d*_*x*_	*q*_*x*_	*L*_*x*_	*T*_*x*_	*e*_*x*_	*lnl*_*x*_	*K*_*x*_
**1**	1	76.9	-230.8	-3000.00	192.31	4730.77	61.50	4.34	-1.39
**2**	4	307.7	76.9	250.00	269.23	4538.46	14.75	5.73	0.29
**3**	3	230.8	-76.9	-333.33	269.23	4269.23	18.50	5.44	-0.29
**4**	4	307.7	0.0	0.00	307.69	4000.00	13.00	5.73	0.00
**5**	4	307.7	-615.4	-2000.00	615.38	3692.31	12.00	5.73	-1.10
**6**	12	923.1	307.7	333.33	769.23	3076.92	3.33	6.83	0.41
**7**	8	615.4	-384.6	-625.00	807.69	2307.69	3.75	6.42	-0.49
**8**	13	1000.0	538.5	538.46	730.77	1500.00	1.50	6.91	0.77
**9**	6	461.5	76.9	166.67	423.08	769.23	1.67	6.13	0.18
**10**	5	384.6	307.7	800.00	230.77	346.15	0.90	5.95	1.61
**11**	1	76.9	76.9	1000.00	38.46	115.38	1.50	4.34	-
**12**	0	0.0	0.0	-	0.00	76.92	-	-	-
**13**	0	0.0	0.0	-	0.00	76.92	-	-	-
**14**	0	0.0	0.0	-	0.00	76.92	-	-	-
**15**	0	0.0	0.0	-	0.00	76.92	-	-	-
**16**	0	0.0	0.0	-	0.00	76.92	-	-	-
**17**	0	0.0	-76.9	-	38.46	76.92	-	-	-
**18**	1	76.9	76.9	1000.00	38.46	38.46	0.50	4.34	-

**Table 3:** Time-specific life table of *P*. *flavus* LM population.

*X*(year)	*a*_*x*_	*l*_*x*_	*d*_*x*_	*q*_*x*_	*L*_*x*_	*T*_*x*_	*e*_*x*_	*lnl*_*x*_	*K*_*x*_
**1**	1	100.0	-100.0	-1000.00	150.00	4250.00	42.50	4.61	-0.69
**2**	2	200.0	0.0	0.00	200.00	4100.00	20.50	5.30	0.00
**3**	2	200.0	-100.0	-500.00	250.00	3900.00	19.50	5.30	-0.41
**4**	3	300.0	0.0	0.00	300.00	3650.00	12.17	5.70	0.00
**5**	3	300.0	-700.0	-2333.33	650.00	3350.00	11.17	5.70	-1.20
**6**	10	1000.0	500.0	500.00	750.00	2700.00	2.70	6.91	0.69
**7**	5	500.0	-500.0	-1000.00	750.00	1950.00	3.90	6.22	-0.69
**8**	10	1000.0	700.0	700.00	650.00	1200.00	1.20	6.91	1.20
**9**	3	300.0	200.0	666.67	200.00	550.00	1.83	5.70	1.10
**10**	1	100.0	-100.0	-1000.00	150.00	350.00	3.50	4.61	-0.69
**11**	2	200.0	200.0	1000.00	100.00	200.00	1.00	5.30	-
**12**	0	0.0	0.0	-	0.00	100.00	-	-	-
**13**	0	0.0	0.0	-	0.00	100.00	-	-	-
**14**	0	0.0	0.0	-	0.00	100.00	-	-	-
**15**	0	0.0	0.0	-	0.00	100.00	-	-	-
**16**	0	0.0	0.0	-	0.00	100.00	-	-	-
**17**	0	0.0	0.0	-	0.00	100.00	-	-	-
**18**	0	0.0	-100.0	-	50.00	100.00	-	-	-
**19**	1	100.0	100.0	1000.00	50.00	50.00	0.50	4.61	-

**Table 4:** Time-specific life table of *P*. *flavus* BM population.

*X(year)*	*a*_*x*_	*l*_*x*_	*d*_*x*_	*q*_*x*_	*L*_*x*_	*T*_*x*_	*e*_*x*_	*lnl*_*x*_	*K*_*x*_
**1**	0	0.00	-666.67	-	333.33	5583.33	-	0.00	-6.50
**2**	4	666.67	333.33	500.00	500.00	5333.33	8.00	6.50	0.69
**3**	2	333.33	-333.33	-1000.00	500.00	4833.33	14.50	5.81	-0.69
**4**	4	666.67	-333.33	-500.00	833.33	4500.00	6.75	6.50	-0.41
**5**	6	1000.00	333.33	333.33	833.33	3916.67	3.92	6.91	0.41
**6**	4	666.67	333.33	500.00	500.00	3166.67	4.75	6.50	0.69
**7**	2	333.33	0.00	0.00	333.33	2750.00	8.25	5.81	0.00
**8**	2	333.33	-333.33	-1000.00	500.00	2500.00	7.50	5.81	-0.69
**9**	4	666.67	500.00	750.00	416.67	2000.00	3.00	6.50	1.39
**10**	1	166.67	0.00	0.00	166.67	1583.33	9.50	5.12	0.00
**11**	1	166.67	166.67	1000.00	83.33	1416.67	8.50	5.12	-
**12**	0	0.00	0.00	-	0.00	1333.33	-	-	-
**13**	0	0.00	0.00	-	0.00	1333.33	-	-	-
**14**	0	0.00	-166.67	-	83.33	1416.67	-	-	-
**15**	1	166.67	166.67	1000.00	83.33	1416.67	8.50	5.12	-
**16**	0	0.00	0.00	-	0.00	1333.33	-	-	-
**17**	0	0.00	0.00	-	0.00	1333.33	-	-	-
**18**	0	0.00	-166.67	-	83.33	1333.33	-	-	-
**19**	1	166.67	0.00	0.00	166.67	1416.67	8.50	5.12	0.00
**20**	1	166.67	0.00	0.00	166.67	1416.67	8.50	5.12	0.00
**21**	1	166.67	166.67	1000.00	83.33	1250.00	7.50	5.12	-
**22**	0	0.00	0.00	-	0.00	1166.67	-	-	-
**23**	0	0.00	-333.33	-	166.67	1166.67	-	-	-
**24**	2	333.33	166.67	500.00	250.00	1000.00	3.00	5.81	0.69
**25**	1	166.67	166.67	1000.00	83.33	750.00	4.50	5.12	-
**26**	0	0.00	-166.67	-	83.33	666.67	-	-	-
**27**	1	166.67	166.67	1000.00	83.33	583.33	3.50	5.12	-
**28**	0	0.00	0.00	-	0.00	500.00	-	-	-
**29**	0	0.00	0.00	-	0.00	500.00	-	-	-
**30**	0	0.00	0.00	-	0.00	500.00	-	-	-
**31**	0	0.00	0.00	-	0.00	500.00	-	-	-
**32**	0	0.00	0.00	-	0.00	500.00	-	-	-
**33**	0	0.00	-166.67	-	83.33	500.00	-	-	-
**34**	1	166.67	166.67	1000.00	83.33	416.67	2.50	5.12	-
**35**	0	0.00	0.00	-	0.00	333.33	-	-	-
**36**	0	0.00	0.00	-	0.00	333.33	-	-	-
**37**	0	0.00	0.00	-	0.00	333.33	-	-	-
**38**	0	0.00	-333.33	-	166.67	333.33	-	-	-
**39**	2	333.33	333.33	1000.00	166.67	166.67	0.50	5.81	5.81

As shown in Tables 1–3, negative mortality (*q*_*x*_) is common and similar in the three populations, and the frequency of appearance was 50% (WM population), 41.7% (LM population) and 52.6% (BM population). Viewed from age class divisions, negative mortality was widely distributed across every age stage but is more obviously distributed in the seedling and aging stages and less so in the middle stage, especially when the plants are just reaching puberty.

### 3.3 Age pyramid, survival curve and mortality curve

#### 3.3.1 Age pyramid

**Fig 2:**
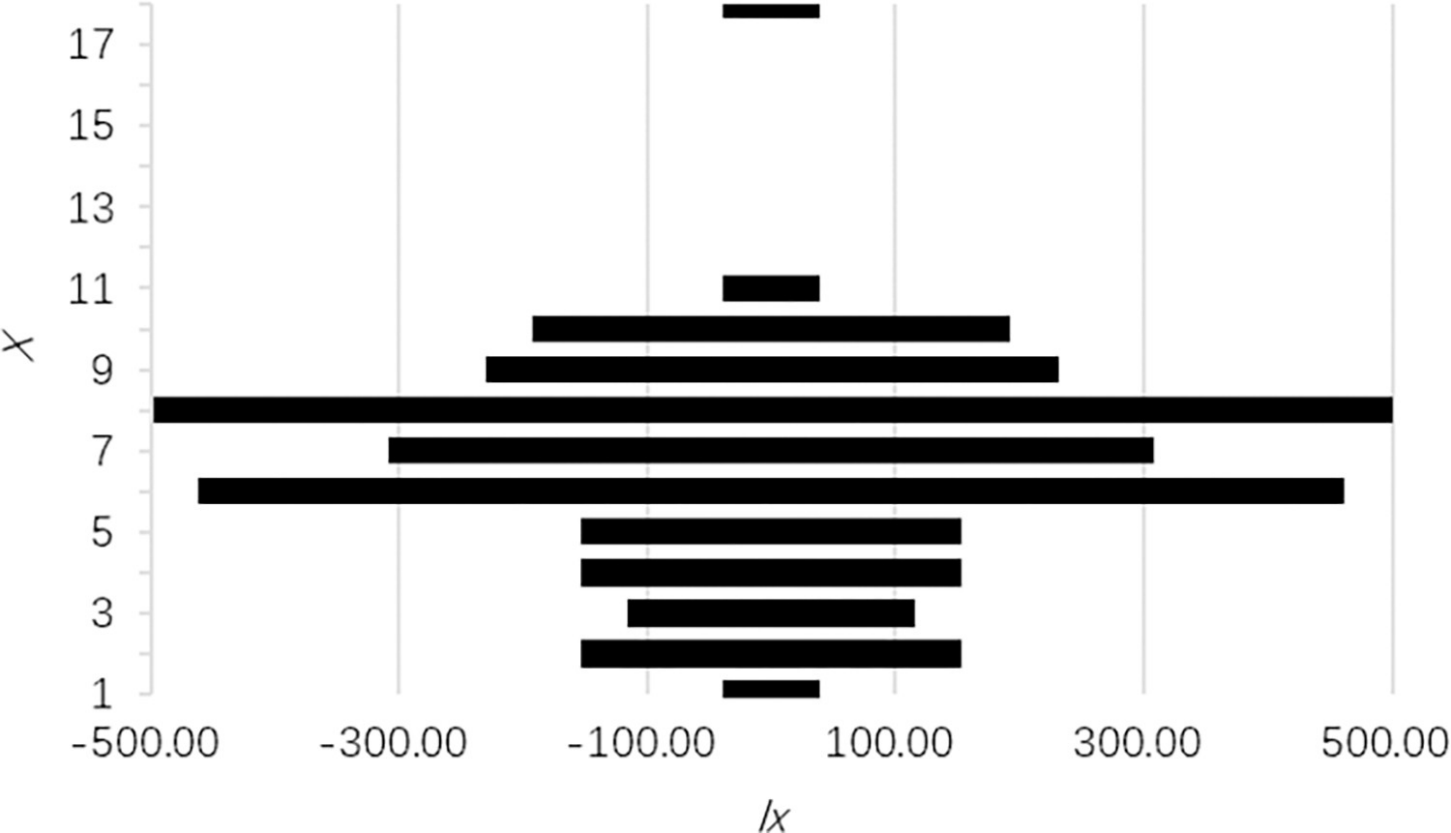
Age pyramid of *P*. *flavus* WM population.

**Fig 3:**
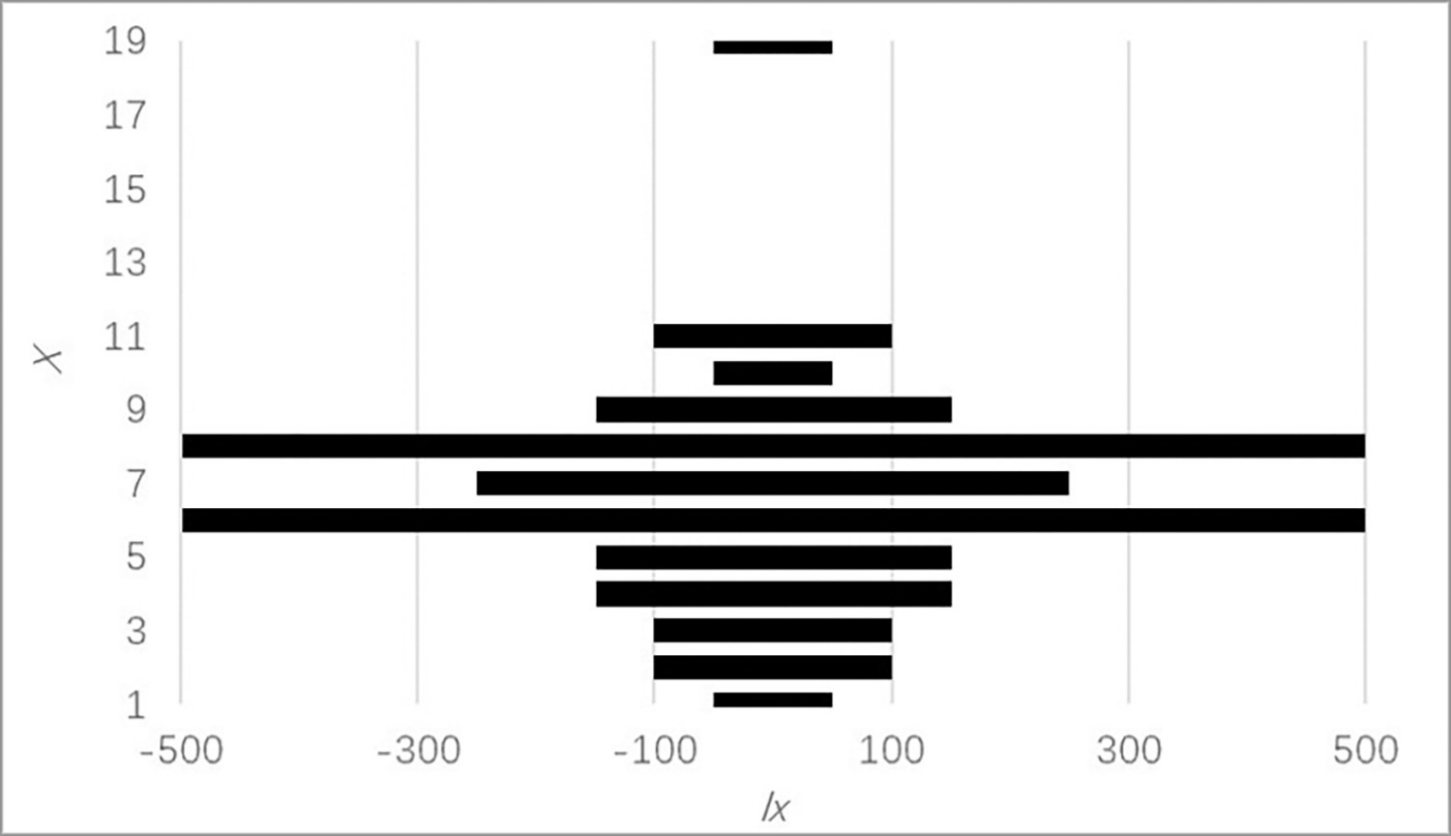
Age pyramid of *P*. *flavus* LM population.

**Fig 4:**
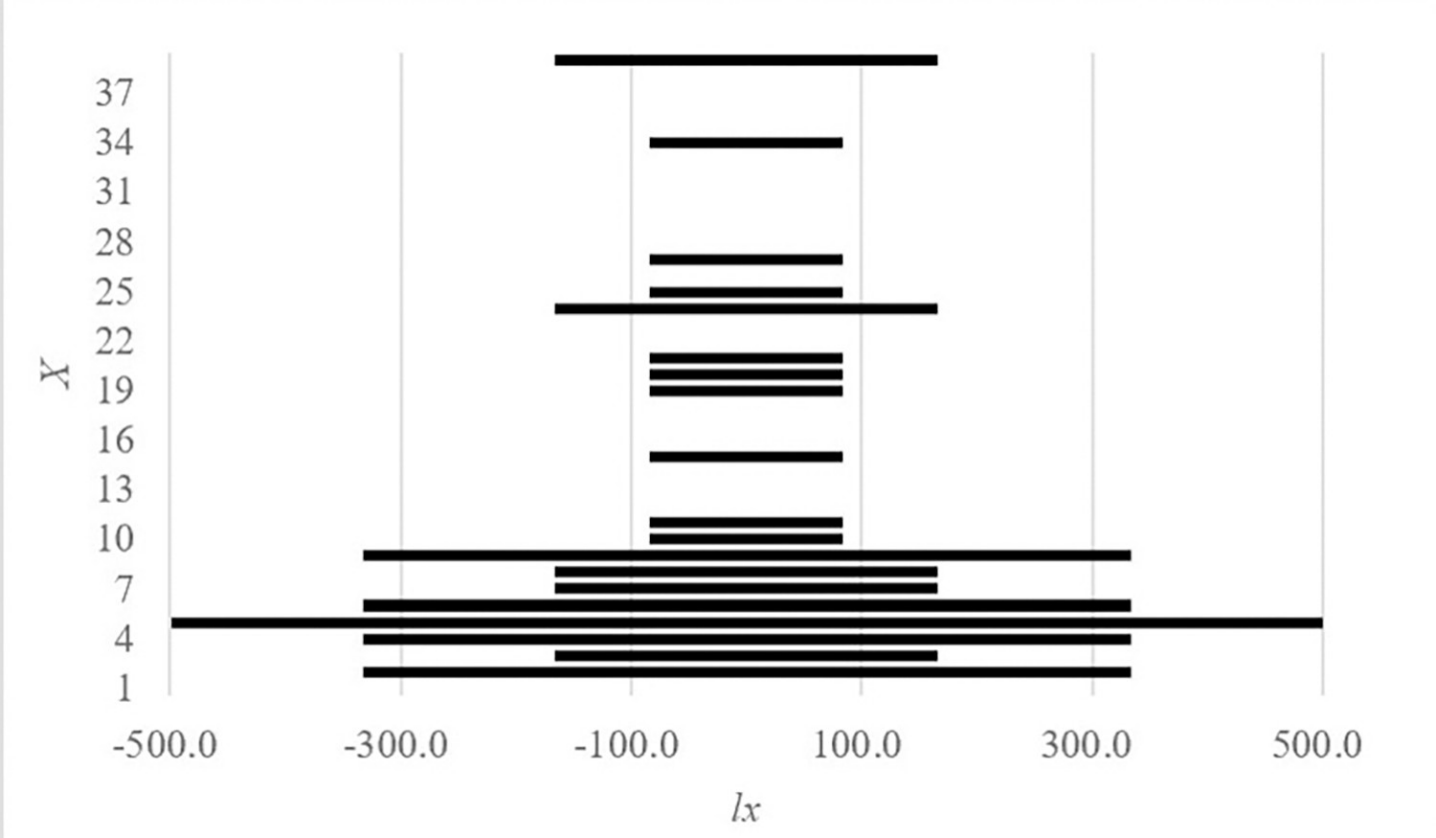
Age pyramid of *P*. *flavus* BM population.

The WM and LM populations have narrower age spans and similar age structures, and as Figs 2 and 3 show, their age pyramids are spindly. The BM population has a wider age span and more significant abruption, and as shown in Fig 4, its age pyramid is roughly bell-shaped and is widest at the bottom and diminishes upward. Therefore, the WM and LM populations are decreasing populations with scarce seedlings. Although most individuals in this kind of population are fertile, it is dangerous to continue lacking seedling recruitment. The BM population has equal numbers of adolescents and adults, which is characteristic of a stable population. In addition, the individuals in these populations were unevenly distributed across age classes, which means the occurrence of unusual deaths. This conclusion is similar to what we found in the time-specific life tables.

#### 3.3.2 Survival curve and mortality curve

**Fig 5:**
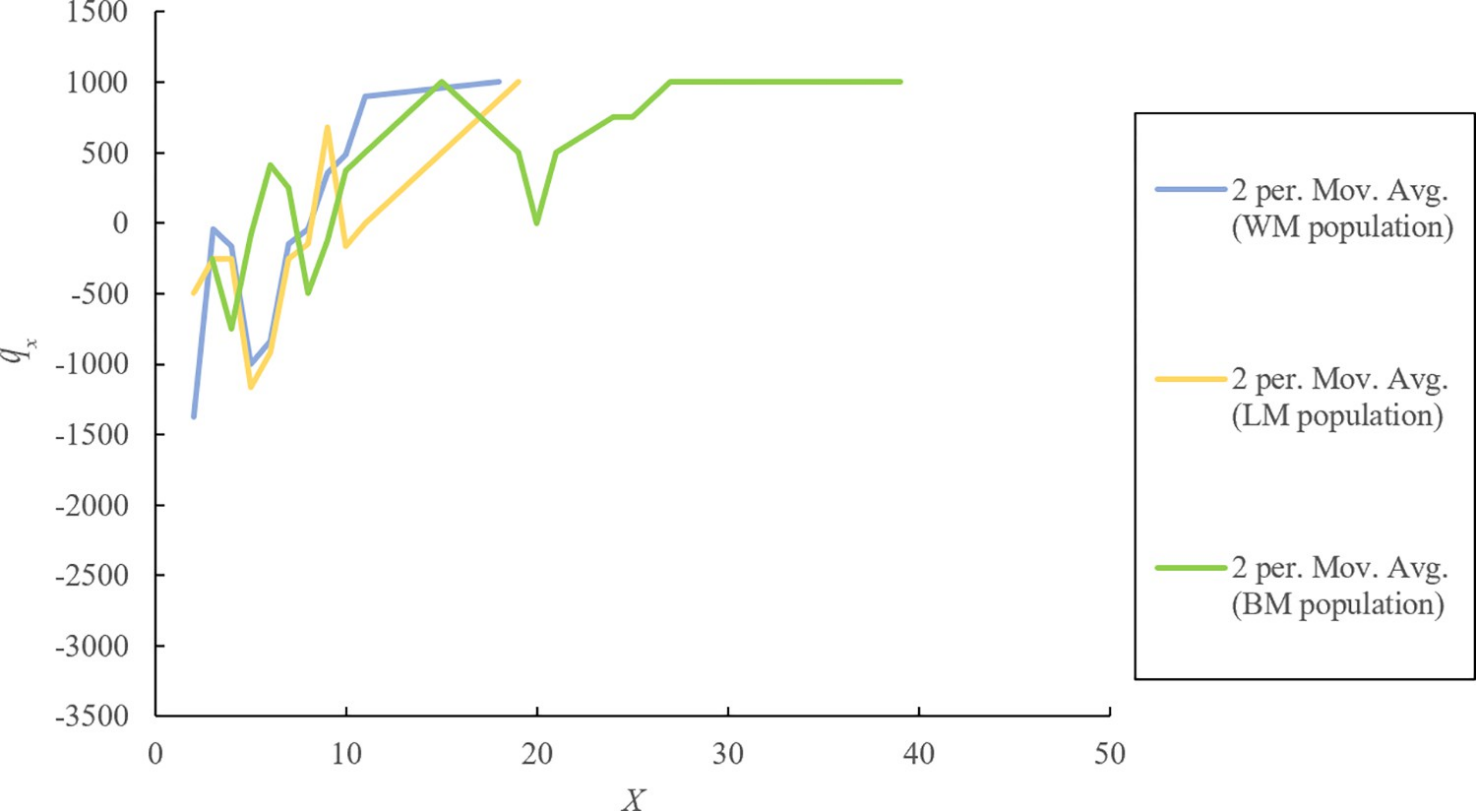
Survival curves of three *P*. *flavus* populations.

**Fig 6:**
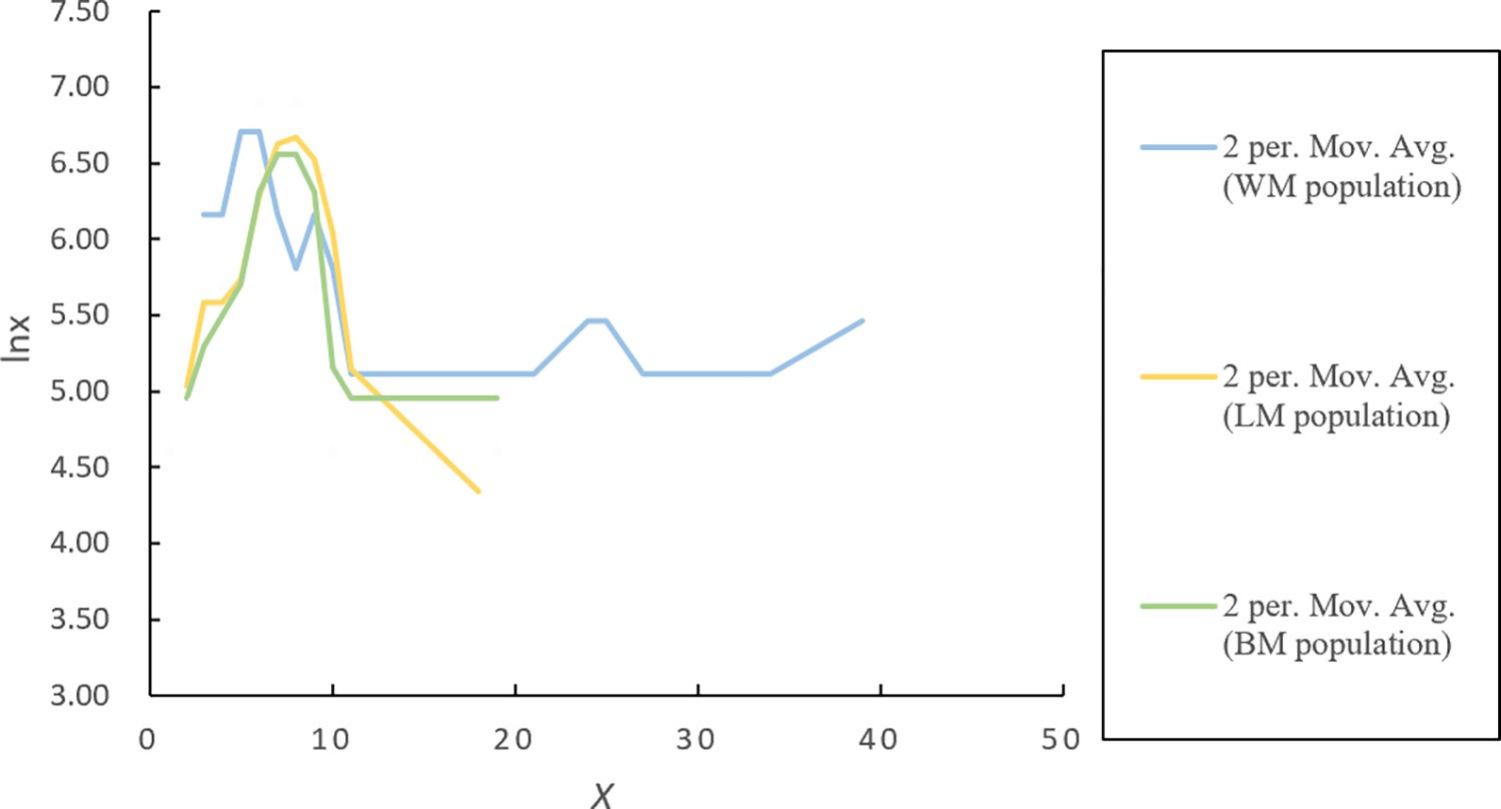
Mortality curves of three *P*. *flavus* populations.

By analyzing the survival curves (Fig 5) and mortality curves (Fig 6) of three *P*. *flavus* populations, the following conclusions can be drawn: 1) As there is a higher survival rate of seedlings (e.g., individuals in the 1–4 age class) in the BM population than in the other two populations, recruitments in the BM population have a higher possibility of entering the breeding period; 2) The peak survival rate appears in the 5–8 age class, and plants in this stage have just begun to flower, while their highest survival rate causes them to become mainstays of population quantities; 3) In the aging stage, the survival curve of the BM population declines steadily, while other survival curves decline steeply, which causes the quantities of the WM and LM populations to be more dependent on their middle-aged individuals.

According to Fig 6, the mortality of *P*. *flavus* plants as a whole increases with increased age, and the peak appears later in life. From the perspective of single age classes, there are significant differences in mortality between one age class and another, which cause the mortality curve to fluctuate widely. Compared with the BM population, the WM and LM populations have higher death rates in the seedling and aging stages, which means that the living status for these two populations are worse than that for the BM population, which is similar to what we found in the survival curves.

Taken together, wild *P*. *flavus* populations currently face a lack of recruitment and difficulty in seedling survival, and their individuals in the middle age class have high living quality and large quantities and bear the burden of sustaining the populations’ existence. Thus, for the WM and LM populations, sharp decreases in seedling numbers cause decreases in their long-term stability; although the mortality curve and survival curve of the BM population showed jagged changes, the BM population still has higher stability, as its overall trend matches the Deevey Ⅰ curve.

### 3.4 Fertility table parameter analysis

*r*_*0*_ (net reproductive rate) and *r*_*m*_ (intrinsic rate of increase) are calculated from the data in Tables 5–7. The *r*_*0*_ values of the three populations are 0.075 (WM population), 0.081 (LM population) and 1.24 (BM population). The *r*_*m*_ values are -0.336 (WM population), -0.392 (LM population) and 0.07 (BM population).

**Table 5:** Fertility table of *P*. *flavus* WM population.

*X*	*l*_*X*_	*m*_*X*_	*l*_X_*m_*X*_	X*l_*X*_ *m_*X*_
**1**	0.08	-	-	-
**2**	0.31	-	-	-
**3**	0.23	-	-	-
**4**	0.31	-	-	-
**5**	0.31	0.02	0.01	0.03
**6**	0.92	0.02	0.02	0.11
**7**	0.62	0.02	0.01	0.09
**8**	1.00	0.02	0.02	0.16
**9**	0.46	0.02	0.01	0.08
**10**	0.38	0.02	0.01	0.08
**11**	0.08	0.02	0.00	0.02
**12**	0.00	0.02	0.00	0.00
**13**	0.00	0.02	0.00	0.00
**14**	0.00	0.02	0.00	0.00
**15**	0.00	0.02	0.00	0.00
**16**	0.00	0.02	0.00	0.00
**17**	0.00	0.02	0.00	0.00
**18**	0.08	0.02	0.00	0.03

**Table 6:** Fertility table of *P*. *flavus* LM population.

*X*	*l*_*X*_	*m*_*X*_	*l*_*X*_ *m_*X*_	X*l_*X*_ *m_*X*_
**1**	0.10	-	-	-
**2**	0.20	-	-	-
**3**	0.20	-	-	-
**4**	0.30	-	-	-
**5**	0.30	0.02	0.01	0.03
**6**	1.00	0.02	0.02	0.14
**7**	0.50	0.02	0.01	0.08
**8**	1.00	0.02	0.02	0.18
**9**	0.30	0.02	0.01	0.06
**10**	0.10	0.02	0.00	0.02
**11**	0.20	0.02	0.00	0.05
**12**	0.00	0.02	0.00	0.00
**13**	0.00	0.02	0.00	0.00
**14**	0.00	0.02	0.00	0.00
**15**	0.00	0.02	0.00	0.00
**16**	0.00	0.02	0.00	0.00
**17**	0.00	0.02	0.00	0.00
**18**	0.00	0.02	0.00	0.00
**19**	0.10	0.02	0.00	0.04

**Table 7:** Fertility table of *P*. *flavus* BM population.

*X*	*l*_*X*_	*m*_*X*_	*l*_*X*_ *m_*X*_	X*l_*X*_ *m_*X*_
**1**	0.00	-	-	-
**2**	0.67	-	-	-
**3**	0.33	-	-	-
**4**	0.67	-	-	-
**5**	1.00	0.24	0.24	1.44
**6**	0.67	0.24	0.16	0.64
**7**	0.33	0.24	0.08	0.16
**8**	0.33	0.24	0.08	0.16
**9**	0.67	0.24	0.16	0.64
**10**	0.17	0.24	0.04	0.04
**11**	0.17	0.24	0.04	0.04
**12**	0.00	0.24	0.00	0.00
**13**	0.00	0.24	0.00	0.00
**14**	0.00	0.24	0.00	0.00
**15**	0.17	0.24	0.04	0.04
**16**	0.00	0.24	0.00	0.00
**17**	0.00	0.24	0.00	0.00
**18**	0.00	0.24	0.00	0.00
**19**	0.17	0.24	0.04	0.04
**20**	0.17	0.24	0.04	0.04
**21**	0.17	0.24	0.04	0.04
**22**	0.00	0.24	0.00	0.00
**23**	0.00	0.24	0.00	0.00
**24**	0.33	0.24	0.08	0.16
**25**	0.17	0.24	0.04	0.04
**26**	0.00	0.24	0.00	0.00
**27**	0.17	0.24	0.04	0.04
**28**	0.00	0.24	0.00	0.00
**29**	0.00	0.24	0.00	0.00
**30**	0.00	0.24	0.00	0.00
**31**	0.00	0.24	0.00	0.00
**32**	0.00	0.24	0.00	0.00
**33**	0.00	0.24	0.00	0.00
**34**	0.17	0.24	0.04	0.04
**35**	0.00	0.24	0.00	0.00
**36**	0.00	0.24	0.00	0.00
**37**	0.00	0.24	0.00	0.00
**38**	0.00	0.24	0.00	0.00
**39**	0.33	0.24	0.08	0.16

Parameter *r*_*0*_ is the increase multiple per generation, and *r*_*0*_ values below 1 mean that the increase quantity is smaller than the decrease quantity and this population cannot achieve growth in numbers and is in danger of dying out; *r*_*0*_ values greater than 1 mean that this population is showing an upward trend. The *r*_*0*_ values for the WM and LM populations are far less than 1, which means that, in these two populations, the instantaneous birth rate is far less than the instantaneous death rate and the tendency for increase is very weak compared with tendency for decrease. Although the *r*_*0*_ of the BM population is greater than 1, it is close to 1. Therefore, the BM population can slowly maintain its growth under present conditions.

Parameter *r*_*m*_ is similar to *r*_*0*_, but its critical value is 0. As we found in the analysis of parameter *r*_*0*_, the WM and LM populations show downward trends, while the BM population is stable.

### 3.5 Leslie matrix and dynamic quantitative prediction model construction

Based on the investigation of biological characteristics, the average number of seedlings produced by fertile individuals in each population was calculated as parameter *m*_*x*_ (average fertility), and the population density was then simulated separately with a matrix model that combined the time-specific life table and fertility table.

Overall, the three populations in this study are declining populations. Population numbers of the WM and LM populations will sharply decrease according to Tables 9 and 11. The continuous reduction of seedling stage plants and, at the same time, the loss of adults aggravates this situation by reducing seed sources and causing a vicious circle until the populations finally vanish. The BM population is different from the two populations mentioned above; its numbers will increase in the mid-early term and decrease later, as shown in Table 13. Before *N*_*20*_, high survival rates of mid-aged individuals become the cornerstone of population numbers; due to the stable supply of recruits offered by fertile plants, the numbers of the BM population will even increase slightly in a few years. The turning point will appear after *N*_*20*_ when the high quality of living of middle stage-plants cannot offset the downward tendency caused by low fertility, and the BM population may begin to decrease gradually until disappearing completely.

#### 3.5.1 WM population

*m*_*x*_ for the WM population is 0.02. The Leslie matrix model and dynamic quantitative prediction model are shown in Tables 8 and 9. The formula for the dynamic quantitative prediction model is *N*_*t*_ = *N*_*t-1*_- *N*_*t-1*_e^-1.56^, where *N*_*t*_ is the number of individuals at time *t*. As predicted, the WM population will vanish after 18 years.

**Table 8:** Leslie matrix model of *P*. *flavus* WM population.

0.000	0.000	0.000	0.000	0.020	0.017	0.014	0.009	0.009	0.003	0.000	0.000	0.000	0.000	0.000	0.000	0.016	0.000
1.400																	
	1.000																
		1.143															
			2.000														
				1.250													
					1.050												
						0.905									0		
							0.579										
								0.545									
									0.167								
										0.000							
											0.000						
		0										0.000					
													0.000				
														0.000			
															0.000		
																1.000	
																	0.000

**Table 9:** Dynamic quantitative prediction model of *P*. *flavus* WM population.

*X*	*N*_*0*_	*N*_*1*_	*N*_*3*_	*N*_*5*_	*N*_*7*_	*N*_*9*_	*N*_*11*_	*N*_*13*_	*N*_*15*_	*N*_*17*_	*N*_*18*_
**1**	1	1	1	0	0	0	0	0	0	0	0
**2**	4	1	1	1	1	0	0	0	0	0	0
**3**	3	4	1	1	1	0	0	0	0	0	0
**4**	4	3	2	1	1	1	0	0	0	0	0
**5**	4	8	9	2	2	2	1	0	0	0	0
**6**	12	5	9	4	2	2	2	1	1	0	0
**7**	8	13	11	12	2	2	2	1	1	1	0
**8**	13	7	5	8	4	2	2	2	1	1	0
**9**	6	8	7	6	6	1	1	1	1	0	0
**10**	5	1	1	0	1	0	0	0	0	0	0
**11**	1	0	0	0	0	0	0	0	0	0	0
**12**	0	0	0	0	0	0	0	0	0	0	0
**13**	0	0	0	0	0	0	0	0	0	0	0
**14**	0	0	0	0	0	0	0	0	0	0	0
**15**	0	0	0	0	0	0	0	0	0	0	0
**16**	0	0	0	0	0	0	0	0	0	0	0
**17**	0	0	0	0	0	0	0	0	0	0	0
**18**	1	0	0	0	0	0	0	0	0	0	0
**Total**	62	51	46	35	19	10	9	6	4	2	0

#### 3.5.2 LM population

*m*_*x*_ of the LM population is 0.02. The Leslie matrix model and dynamic quantitative prediction model are shown in Tables 10 and 11. The formula for the dynamic quantitative prediction model is *N*_*t*_ = *N*_*t-1*_- *N*_*t-1*_e^0.75^, where *N*_*t*_ is the number of individuals at *t* time. As predicted, the LM population will vanish after 22 years.

**Table 10:** Leslie matrix model of *P*. *flavus* LM population.

0.000	0.000	0.000	0.000	0.027	0.023	0.020	0.007	0.017	0.015	0.000	…	0.000	0.000	0.023	0.000
1.333															
	1.250														
		1.200													
			2.167												
				1.154										0	
					1.000										
						0.867									
							0.308								
								0.750							
									0.667						
										0.000					
	0										…				
												0.000			
													0.000		
														1.000	
															0.000

**Table 11:** Dynamic quantitative prediction model of *P*. *flavus* LM population.

*X*	*N*_*0*_	*N*_*1*_	*N*_*3*_	*N*_*5*_	*N*_*7*_	*N*_*9*_	*N*_*11*_	*N*_*13*_	*N*_*15*_	*N*_*17*_	*N*_*19*_	*N*_*21*_	*N*_*22*_
**1**	1	1	1	1	0	0	0	0	0	0	0	0	0
**2**	2	1	1	1	1	0	0	0	0	0	0	0	0
**3**	2	3	1	1	1	1	0	0	0	0	0	0	0
**4**	3	2	2	1	1	1	1	0	0	0	0	0	0
**5**	3	7	7	2	2	2	1	1	1	1	0	0	0
**6**	10	3	6	5	3	3	2	1	1	1	1	0	0
**7**	5	10	8	8	3	3	3	2	1	1	1	1	0
**8**	10	4	3	5	4	2	2	2	1	1	1	1	0
**9**	3	3	3	2	2	1	1	1	0	0	0	0	0
**10**	1	2	1	1	1	1	1	1	0	0	0	0	0
**11**	2	1	2	1	1	1	0	0	0	0	0	0	0
**12**	0	0	0	0	0	0	0	0	0	0	0	0	0
**13**	0	0	0	0	0	0	0	0	0	0	0	0	0
**14**	0	0	0	0	0	0	0	0	0	0	0	0	0
**15**	0	0	0	0	0	0	0	0	0	0	0	0	0
**16**	0	0	0	0	0	0	0	0	0	0	0	0	0
**17**	0	0	0	0	0	0	0	0	0	0	0	0	0
**18**	0	0	0	0	0	0	0	0	0	0	0	0	0
**19**	1	0	0	0	0	0	0	0	0	0	0	0	0
**Total**	43	37	35	28	19	15	11	8	4	4	3	2	0

#### 3.5.3 BM population

*m*_*x*_ of the BM population is 0.24. The Leslie matrix model and dynamic quantitative prediction model are shown in Tables 12 and 13. The formula of the dynamic quantitative prediction model is *N*_*t*_ = *N*_*t-1*_- *N*_*t-1*_e^0.71^, where *N*_*t*_ is the number of individuals at *t* time. As predicted, the BM population will vanish after 40 years.

**Table 12:** Leslie matrix model of *P*. *flavus* BM population.

0.000	0.000	0.000	0.000	0.144	0.161	0.360	0.199	0.096	0.120	…	0.360	0.079	0.240	0.240	…	0.240	0.000
1.500																	
	1.000																
		1.670															
			1.000														
				0.600													
					0.670											0	
						1.500											
							0.830										
								0.400									
									0.500								
										…							
	0										1.500						
												0.330					
													1.000				
														1.000			
															…		
																1.000	
																	0.000

**Table 13:** Dynamic quantitative prediction model of *P*. *flavus* BM population.

*X*	*N*_*0*_	*N*_*5*_	*N*_*10*_	*N*_*15*_	*N*_*20*_	*N*_*25*_	*N*_*30*_	*N*_*35*_	*N*_*39*_	*N*_*40*_
**1**	0	1	3	3	2	1	1	0	0	0
**2**	4	3	2	4	6	0	1	1	0	0
**3**	2	4	1	2	4	0	1	0	0	0
**4**	4	8	2	3	4	2	1	0	0	0
**5**	6	10	4	7	4	5	2	0	0	0
**6**	4	0	1	4	4	3	1	1	1	0
**7**	2	3	2	2	3	4	0	1	0	0
**8**	2	2	4	1	2	4	0	1	0	0
**9**	4	2	4	1	2	2	1	1	0	0
**10**	1	1	2	1	1	1	1	0	0	0
**11**	1	1	0	0	1	1	0	0	0	0
**12**	0	0	0	0	0	0	0	0	0	0
**13**	0	0	0	0	0	0	0	0	0	0
**14**	0	0	0	0	0	0	0	0	0	0
**15**	1	0	0	0	0	0	0	0	0	0
**16**	0	0	0	0	0	0	0	0	0	0
**⋮**	**⋮**	**⋮**	**⋮**	**⋮**	**⋮**	**⋮**	**⋮**	**⋮**	**⋮**	**⋮**
**39**	2	0	0	0	0	0	0	0	0	0
**Total**	41	35	25	28	33	23	9	5	1	0

## 4 Discussion

### 4.1 The formation of a negative mortality phenomenon

In the analysis of time-specific life tables, we found that negative mortality (*q*_*x*_) was common in every period of each population. This is unusual. As a kind of ecological study method that works backward from spatial sequence to temporal sequence, it is usually used to study long-lived species (their complete life history is hard to monitor) whose ages can be accurately evaluated. This is why specific-time life tables are generally used for tree research [32]. *P*. *flavus* plants have age indicators, and the age of the oldest plants in three populations was 39 years, so this method applies to *P*. *flavus*. In recent years, wild populations of *Cypripedium lentiginosum* [30], *Cymbidium sinense* [8], *Pleione formosana* [29], *Dendrobium sinense* [27] and other orchids have been studied by life table methods. In the research of *D*.*sinense*, the negative mortality issue was raised and was explained by “unusual deaths”. CHEN used data-smoothing techniques to correct the negative death rate problem in his research on *Cinnamomum micranthum* [33]. This technique is widely recognized and has been applied in some studies in recent years [34, 35]. Throughout the studies that used data-smoothing techniques, their objects of study not only had far greater quantities than this study but also had more balanced distributions of age classes; these are probably the characteristics that made them suitable for this technique. This method is more applicable for repairing errors that do not affect overall trends, so this study was excluded from its application. We posed two hypotheses regarding the high frequency of negative mortality: 1) seedling numbers have fluctuated and 2) *P*. *flavus* plants have been overcollected.

#### 4.1.1 Speculation on the cause of recruitment fluctuations

The WM and LM populations only produced 1 recruitment last year, while the BM population had none. This is far below the maximum number of survivors in each population. *P*. *flavus* plants use both sexual and asexual reproduction as a kind of clonal plant. Clonal plants produce ramets by breaking the connecting structures between the ramet and genet [36]. Most of the time, one *P*. *flavus* individual will produce one or more pseudobulb per year but these potential ramets have difficulty separating from their genets because the short fleshy stems that link them together are hard to break or rot under natural conditions. From another perspective, the long-term existence of connecting structures may decrease ramet mortality by nutrition storage and absorption of resources [37]. Therefore, it is shown in this study that asexual reproduction of *P*. *flavus* plants has more value for the extension of mother plants, stabilization of population numbers, and accumulation of dry matter, rather than the production of new individuals. The increase in seedling numbers mainly depends on sexual reproduction.

According to our survey of persistent fruits, there are significant differences between quantities, and each population had ripe and dehiscent fruits. This evidence suggests that *P*. *flavus* plants can complete their sexual reproduction process without any apparent difficulties. Additionally, Wang et al. studied the aseptic germination of *P*. *flavus* plants, and they proved that the seed germination rate could be above 70% and that the rooting rate could be above 90% without special treatment under experimental conditions [22]. It can be inferred from this report that *P*. *flavus* seeds have no internal structural barriers to germination when compared with other orchids that do have difficulty germinating, such as *Paphiopedilum*. Our study supports the notion that germination of *P*. *flavus* seeds is highly dependent on environmental suitability and on the availability of mycorrhiza, which means that the seedling complements of *P*. *flavus* populations in the wild are easily affected by environmental change, thus causing fluctuations in recruitment quantities.

#### 4.1.2 The effect of wild harvesting

Wild harvesting of orchids, such as *P*. *flavus*, is assumed to be unsustainable because their life story characteristics cause many orchids to be vulnerable to disturbances [38]. Particularly in the flowering period, their large, bright, yellow flowers may attract more attention from passers-by and this increases the risk of individual blossoms been harvested. This not only directly causes the loss of adult individuals but also indirectly causes the loss of potential seedlings. Lin et al. believe that maintaining human interference at a moderate level can provide more opportunities for other plants to settle in the community [39]. A similar conclusion was drawn by Niu Li-qin in her essay, and she believes that moderate human disturbance is beneficial for increasing plant species richness [40]. Both of their studies are based on the idea that species that have been disturbed can still maintain their growth in population numbers, while the overall trend of *P*. *flavus* wild populations has been falling. Therefore, protecting these populations should come first, as wild harvesting still does more harm than good.

### 4.2 Development gaps between three *P*. *flavus* populations

Compared with the BM population, the population structure of the LM population is more similar to that of the WM population in the southern subtropical region. The LM and BM populations are both in the middle subtropical region. Therefore, the development of differences between these three populations is not entirely dependent on natural environmental factors but also influenced by internal factors and human interference.

On the one hand, nutrient accumulations by plants are important for reproductive success. The phenomenon that fruit and seedling production in orchids differs between different populations within species has also been found in other research [41]. Compared with other populations, the BM population is significantly higher than the others in the number of age classes and average age, which means that plants in the BM population made fuller matter preparation. In the statistics of persistent fruits, we found that the output due to sexual reproduction in the BM population was highest in the three populations, as the fruit average of the BM population (2.63) was far greater than that for the others (e.g., 0.05 for the WM population and 0.26 for the LM population). Therefore, for the case of slight differences in other natural environmental factors, it is speculated that the sexual reproduction activity of *P*. *flavus* is likely to be positively correlated with the degree of material accumulation. On the other hand, the WM population is near a footpath in Sandiejing Forest Park, so its habitat highly overlaps with areas of human activity; the distance between the LM population and a local tea garden is less than 1 km; the BM population is located in an inaccessible valley. Based on accessibility analysis, the WM population is the most likely to be disturbed by humans, the LM population is second most likely, and the BM population is least likely to be disturbed.

### 4.3 Reproduction strategies of *P*. *flavus*

In a study of *Trias verrucosa*, LIU et al. found that this orchid has a reproduction strategy of strengthening asexual reproduction and weakening sexual reproduction as a response to the low rewards of sexual reproduction [26]. In their matrix analyses, Miguel Franco and Jonathan Silvertown also suggested that seed production and seedling recruitment are secondary to the long-term viability of populations of long-lived plant species, including orchids [42]. We assume that a similar phenomenon occurs in *P*. *flavus* populations. Although there is no barrier to its sexual reproduction, the development focus of young populations may shift to asexual reproduction to ensure the survival of those populations. The adult stages of orchids are profoundly different from the juvenile stages. Jacquemyn et al. suggested that adult orchids generally exhibit much lower annual mortality rates and, in many species, have the potential to achieve long lifespans [43]. Therefore, investment in asexual reproduction appears to be more worthwhile for young populations to use less energy and gain more profits [43]. After long-term accumulation of nutrients, mature populations are more competitive in their surroundings, which gives them the ability to continue surviving while pursuing sexual reproduction to seek more potential opportunities for future development and expansion [26]. Zhang suggested that most resources should be allocated toward sexual reproduction in habitats with fluctuating environmental conditions and strong competition [44]. Obviously, the development of this reproduction strategy is related to the relatively stable growing environment of *P*. *flavus* populations.

### 4.4 Protective and restoring measures

As we found in this study, the survival prognosis for wild *P*. *flavus* populations is poor; the WM and LM populations are at risk of dying out, and the BM population can barely maintain stable numbers. Based on the above analysis, the fundamental cause of the decline of *P*. *flavus* wild populations lies in recruitment shortages caused by low fertility, while the loss of adults triggered by human harvesting aggravates this difficult position and speeds up the decrease in the number of individuals. The seedling shortage issue is probably due to a combination of *P*. *flavus* own reproduction strategies and the germination difficulties that are commonly seen in orchids. Therefore, ensuring survival of adults is a critical part of promoting the recovery and renewal of *P*. *flavus* populations. According to the analysis of endangering mechanisms, it is suggested that human activities around *P*. *flavus* populations be strictly limited to reduce over-collection and habitat breakage. These populations should be revisited every year to confirm their status so that we can conduct ex situ conservation in time.

## Supporting information

S1 Raw images (PDF)

S1 Table Original population dynamic data. (DOCX)
