# Supplementary figures and images for: Population dynamics of Phaius flavus in southeast China: Reproductive strategies and plants conservation
Source: PLoS One. 2022 Aug 15;17(8):e0272929. doi: 10.1371/journal.pone.0272929 (PMC9377627; doi:10.1371/journal.pone.0272929)

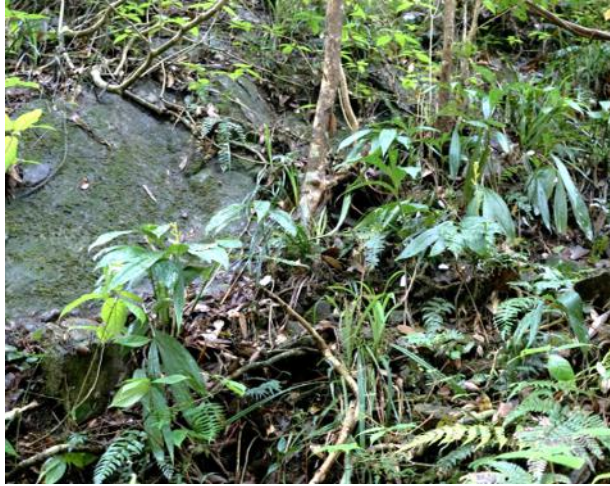

A. Habitat;

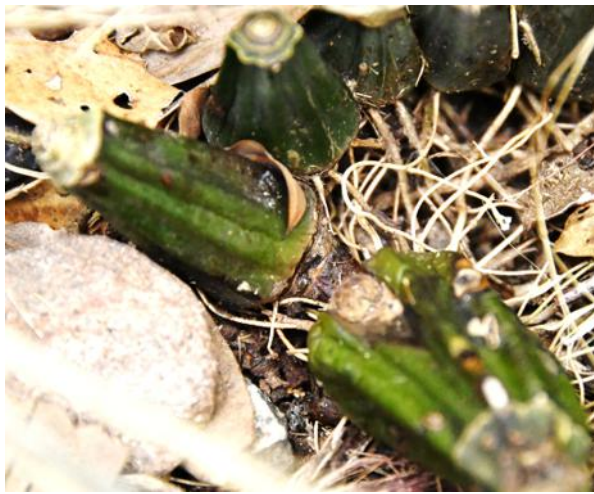

B. pseudobulbs and fleshy stems;

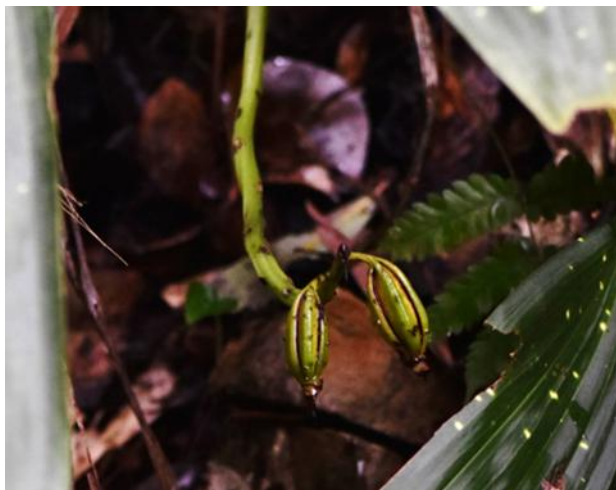

C. persistent dehiscent fruits ;

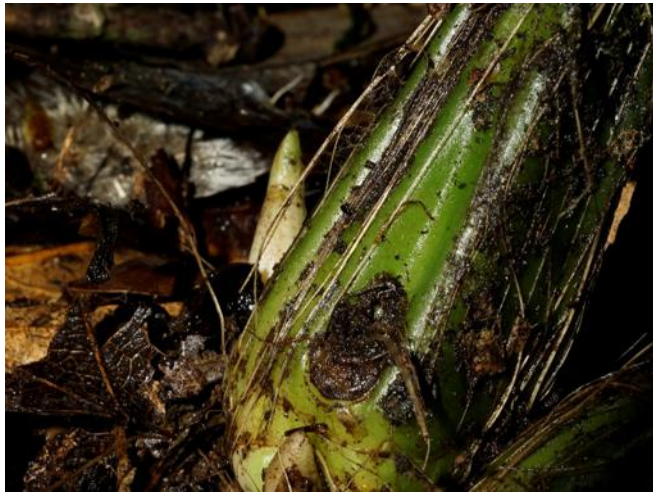

D. a budding bud

Supplement: S1 Raw images — (PDF) [file pone.0272929.s001.pdf]
