# Supplementary material for: Population dynamics of Phaius flavus in southeast China: Reproductive strategies and plants conservation
Source: PLoS One. 2022 Aug 15;17(8):e0272929. doi: 10.1371/journal.pone.0272929 (PMC9377627; doi:10.1371/journal.pone.0272929)
Supplement: S1 Table — (DOCX) [file pone.0272929.s002.docx]

| Wuhu Mountain Population | | | |
| --- | --- | --- | --- |
| Number | All pseudobulb | Leafed pseudobulb | New bud |
| 1 | 7 | 2 | 1 |
| 2 | 6 | 2 | 0 |
| 3 | 6 | 2 | 0 |
| 4 | 6 | 2 | 0 |
| 5 | 6 | 2 | 0 |
| 6 | 5 | 2 | 1 |
| 7 | 10 | 1 | 1 |
| 8 | 6 | 1 | 1 |
| 9 | 8 | 2 | 1 |
| 10 | 9 | 1 | 0 |
| 11 | 8 | 2 | 1 |
| 12 | 7 | 2 | 1 |
| 13 | 8 | 1 | 1 |
| 14 | 8 | 2 | 1 |
| 15 | 9 | 1 | 1 |
| 16 | 6 | 2 | 1 |
| 17 | 6 | 2 | 0 |
| 18 | 4 | 2 | 1 |
| 19 | 2 | 1 | 1 |
| 20 | 7 | 2 | 0 |
| 21 | 8 | 2 | 1 |
| 22 | 8 | 2 | 0 |
| 23 | 11 | 2 | 1 |
| 24 | 9 | 2 | 1 |
| 25 | 10 | 2 | 1 |
| 26 | 8 | 1 | 1 |
| 27 | 7 | 2 | 1 |
| 28 | 6 | 2 | 1 |
| 29 | 3 | 1 | 1 |
| 30 | 2 | 1 | 1 |
| 31 | 5 | 1 | 1 |
| 32 | 5 | 1 | 1 |
| 33 | 2 | 1 | 1 |
| 34 | 8 | 2 | 1 |
| 35 | 10 | 2 | 1 |
| 36 | 6 | 1 | 1 |
| 37 | 8 | 3 | 1 |
| 38 | 3 | 1 | 1 |
| 39 | 18 | 3 | 1 |
| 40 | 3 | 1 | 1 |
| 41 | 6 | 2 | 1 |
| 42 | 9 | 2 | 1 |
| 43 | 7 | 2 | 1 |
| 44 | 8 | 2 | 1 |
| 45 | 7 | 2 | 1 |
| 46 | 10 | 2 | 1 |
| 47 | 8 | 2 | 1 |
| 48 | 3 | 2 | 1 |
| 49 | 4 | 2 | 1 |
| 50 | 6 | 2 | 1 |
| 51 | 5 | 1 | 1 |
| 52 | 6 | 2 | 1 |
| 53 | 3 | 1 | 1 |
| 54 | 10 | 2 | 1 |
| 55 | 7 | 1 | 1 |
| 56 | 7 | 2 | 1 |
| 57 | 8 | 3 | 1 |
| 58 | 2 | 2 | 1 |
| 59 | 4 | 2 | 1 |
| 60 | 4 | 1 | 1 |
| 61 | 8 | 2 | 1 |
| 62 | 7 | 2 | 1 |

| Luohan Mountain Population | | | |
| --- | --- | --- | --- |
| Number | All pseudobulb | Leafed pseudobulb | New bud |
| 1 | 8 | 2 | 1 |
| 2 | 2 | 2 | 1 |
| 3 | 5 | 1 | 1 |
| 4 | 3 | 2 | 2 |
| 5 | 6 | 2 | 1 |
| 6 | 7 | 2 | 1 |
| 7 | 5 | 2 | 1 |
| 8 | 11 | 1 | 1 |
| 9 | 19 | 8 | 2 |
| 10 | 2 | 2 | 2 |
| 11 | 1 | 1 | 1 |
| 12 | 8 | 1 | 1 |
| 13 | 7 | 1 | 0 |
| 14 | 3 | 2 | 0 |
| 15 | 6 | 3 | 0 |
| 16 | 9 | 2 | 0 |
| 17 | 8 | 2 | 1 |
| 18 | 9 | 2 | 1 |
| 19 | 8 | 2 | 0 |
| 20 | 4 | 2 | 1 |
| 21 | 8 | 3 | 1 |
| 22 | 6 | 2 | 1 |
| 23 | 6 | 3 | 1 |
| 25 | 6 | 3 | 1 |
| 26 | 6 | 2 | 1 |
| 27 | 11 | 2 | 1 |
| 28 | 8 | 2 | 0 |
| 29 | 5 | 2 | 0 |
| 30 | 8 | 1 | 1 |
| 31 | 6 | 1 | 1 |
| 32 | 4 | 1 | 1 |
| 33 | 7 | 2 | 1 |
| 34 | 6 | 2 | 1 |
| 35 | 10 | 2 | 1 |
| 36 | 4 | 2 | 1 |
| 37 | 6 | 2 | 1 |
| 38 | 6 | 1 | 1 |
| 39 | 7 | 1 | 1 |
| 40 | 8 | 1 | 1 |
| 41 | 7 | 2 | 1 |
| 42 | 8 | 2 | 1 |
| 43 | 9 | 1 | 1 |
| 44 | 8 | 2 | 1 |
| 50 | 4 | 1 | 1 |

| Beikengding Mountain Population | | | |
| --- | --- | --- | --- |
| Number | All pseudobulb | Leafed pseudobulb | New bud |
| A1 | 19 | 4 | 1 |
| A2 | 10 | 4 | 2 |
| A3 | 9 | 3 | 1 |
| A4 | 9 | 2 | 1 |
| A5 | 5 | 1 | 1 |
| A6 | 7 | 2 | 1 |
| A7 | 9 | 2 | 0 |
| A8 | 11 | 3 | 1 |
| A9 | 15 | 5 | 0 |
| A10 | 6 | 2 | 1 |
| A11 | 4 | 1 | 1 |
| A12 | 2 | 1 | 1 |
| A13 | 6 | 2 | 1 |
| A14 | 8 | 2 | 1 |
| A15 | 7 | 2 | 0 |
| A16 | 6 | 2 | 1 |
| A17 | 9 | 1 | 1 |
| A18 | 5 | 2 | 0 |
| A19 | 2 | 2 | 0 |
| A20 | 5 | 2 | 1 |
| A21 | 2 | 2 | 0 |
| A22 | 2 | 2 | 0 |
| A23 | 4 | 2 | 0 |
| A24 | 5 | 1 | 1 |
| A25 | 3 | 1 | 1 |
| A26 | 4 | 2 | 1 |
| B1 | 24 | 7 | 0 |
| B2 | 20 | 7 | 2 |
| B3 | 24 | 6 | 1 |
| B4 | 2 | 2 | 0 |
| B5 | 21 | 4 | 1 |
| B6 | 34 | 9 | 2 |
| B7 | 6 | 1 | 1 |
| B8 | 3 | 1 | 1 |
| B9 | 5 | 1 | 1 |
| B10 | 27 | 6 | 0 |
| B11 | 39 | 16 | 0 |
| B12 | 8 | 3 | 3 |
| B13 | 25 | 3 | 1 |
| B14 | 39 | 6 | 1 |
| B15 | 5 | 1 | 1 |
